# Synthetic Lagrangian turbulence by generative diffusion models

**DOI:** 10.1038/s42256-024-00810-0

**Published:** 2024-04-17

**Authors:** T. Li, L. Biferale, F. Bonaccorso, M. A. Scarpolini, M. Buzzicotti

**Affiliations:** 1 Dept. of Physics and INFN, University of Rome Tor Vergata, Rome, Italy; 2 Dept. of Industrial Engineering, University of Rome Tor Vergata, Rome, Italy

**Keywords:** Fluid dynamics, Statistical physics

## Abstract

Lagrangian turbulence lies at the core of numerous applied and fundamental problems related to the physics of dispersion and mixing in engineering, biofluids, the atmosphere, oceans and astrophysics. Despite exceptional theoretical, numerical and experimental efforts conducted over the past 30 years, no existing models are capable of faithfully reproducing statistical and topological properties exhibited by particle trajectories in turbulence. We propose a machine learning approach, based on a state-of-the-art diffusion model, to generate single-particle trajectories in three-dimensional turbulence at high Reynolds numbers, thereby bypassing the need for direct numerical simulations or experiments to obtain reliable Lagrangian data. Our model demonstrates the ability to reproduce most statistical benchmarks across time scales, including the fat-tail distribution for velocity increments, the anomalous power law and the increased intermittency around the dissipative scale. Slight deviations are observed below the dissipative scale, particularly in the acceleration and flatness statistics. Surprisingly, the model exhibits strong generalizability for extreme events, producing events of higher intensity and rarity that still match the realistic statistics. This paves the way for producing synthetic high-quality datasets for pretraining various downstream applications of Lagrangian turbulence.

## Abstract (web summary)

Modelling the statistical and geometrical properties of particle trajectories in turbulent flows is key to many scientific and technological applications. Li and colleagues introduce a data-driven diffusion model that can generate high-Reynolds-number Lagrangian turbulence trajectories with statistical properties consistent with those of the training set and even generalize to rare, intense events unseen during training.

## Main

**Fig. 1:**
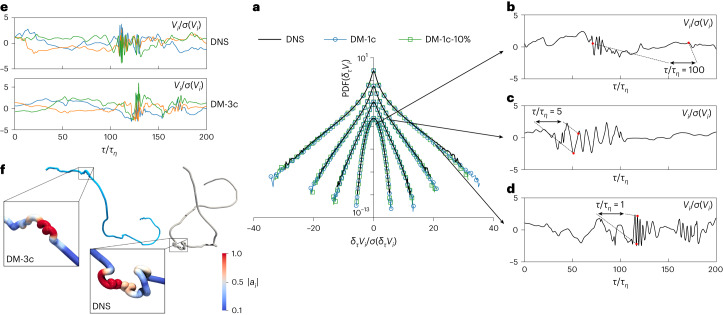
Comparison between DNSs and DMs. **a**, Standardized PDFs of one generic component of the velocity increment, *δ*_*τ*_*V*_*i*_, at *τ*/*τ*_*η*_ = 1, 2, 5, 100 for ground-truth DNS data (black lines), synthetically generated data from DM-1c (blue lines with circles) and that from DM-1c-10% (green lines with squares), a DM-1c model trained with 10% DNS data. PDFs for different *τ* are vertically shifted for the sake of presentation. *σ* is the standard deviation. **b**–**d**, DM-1c trajectories for one generic velocity component with large (**b**), medium (**c**) and small (**d**) time increments, *τ*/*τ*_*η*_ = 100, 5, 1, respectively. **e**, Comparison of 3D trajectories showing small-scale vortex structures for both DNS and DM-3c data, where different curves correspond to the three standardized velocity components *i* = *x*, *y*, *z*. For the DNS, the high oscillatory correlations between the three components are consistent with the presence of strong vortical structures. Similarly, in the case of DM-3c, these correlations can be interpreted as reflecting vortical structures within the hypothetical Eulerian flow. **f**, Examples of 3D trajectories reconstructed from DNS (bottom) and DM-3c (top). Notice in panel **a** the remarkable generalizability properties of our DM data-driven model, able to explore and capture extreme events for velocity fluctuations with far larger intensities than observed in the DNS dataset, represented by much more extended tails, while still maintaining the ground-truth statistics inherent in the training data. Here, the statistics for DM-1c and DM-1c-10% data are derived from 86 and 22 times the number of trajectories in the DNS, respectively. Source data

**Fig. 2:**
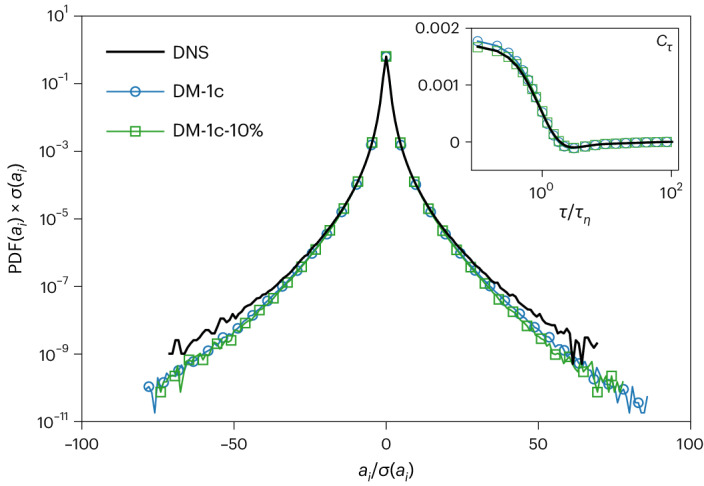
Statistics of acceleration. Standardized PDFs of one generic component of the acceleration, *a*_*i*_, for ground-truth DNS data (black line), synthetically generated data from DM-1c (blue line with circles) and that from DM-1c-10% (green line with squares). Notice the ability of DM-1c to well generalize the statistical trend for rare intense fluctuations never experienced during the training phase with the DNS data. The statistics of the DM-1c and DM-1c-10% data are based on 86 and 22 times the number of trajectories in the DNS, respectively. Inset: acceleration correlation function. Source data

Understanding the statistical and geometrical properties of particles advected by turbulent flows is a challenging problem of utmost importance for modelling, predicting and controlling many applications such as combustion, industrial mixing, pollutant dispersion, quantum fluids, protoplanetary disks accretion, cloud formation and prey–predator dynamics, to cite just a few^1–16^. The main difficulties arise from the vast range of time scales involved, spanning from the longest, *τ*_*L*_, governed by the stirring mechanism, to the shortest, *τ*_*η*_, associated with viscous dissipation and the presence of strong non-Gaussian fluctuations (intermittency). Indeed, the ratio *τ*_*L*_/*τ*_*η*_ is proportional to the Taylor Reynolds number, *R*_*λ*_, a dimensionless measure of the turbulent intensity, varying from a few thousand in laboratory experiments to millions and even larger in atmospheric and astrophysical contexts^17^. Similarly, non-Gaussian fat tails become more pronounced with increasing *R*_*λ*_, resulting in rare-but-intense velocity and acceleration fluctuations of up to 50–60 standard deviations that can be easily measured even in table-top laboratory flows at moderate *R*_*λ*_ (Figs. 1a and 2). Due to the combined influence of long-distance sweeping, multitime fluctuations and small-scale trapping within intense minitornadoes, the problem remains insurmountable from both theoretical and modelling perspectives at the present time.

Over the past 30 years, many different Lagrangian phenomenological models have been proposed, employing various methods such as two-time Ornstein–Uhlembeck stochastic approaches, to capture the dynamics at the two spectrum extremes, *τ*_*L*_, *τ*_*η*_ (refs. ^18,19^) as well as multitime infinite-differentiable processes^20^. Numerous other models have explored with differing degrees of success, including applications to passive scalar fluctuations^21–25^. Moreover, both Markovian and non-Markovian modelization based on multifractal and/or multiplicative models have been employed previously to reproduce certain observed Lagrangian and Eulerian multiscale turbulent features^26–31^; see ref. ^32^ for a recent attempt to combine multifractal scaling and stochastic partial differential equations. However, although all these previous attempts are able to reproduce well some non-trivial features of the turbulent statistics, we still lack a systematic way to generate synthetic trajectories with the correct multiscale statistics over the full range of dynamics encountered in a real turbulent environment, from the large forcing scales, through the intermittent inertial range, to the coupled regime between inertial and dissipative scales^33^.

As a result, new approaches are needed to attack the problem. Machine learning (ML) synthetic data-driven models, including variational autoencoders^34^, generative adversarial networks (GANs)^35^ and, more recently, diffusion models (DMs)^36^, have exhibited remarkable success across diverse fields such as computer vision, audio generation, natural language processing, healthcare and various other domains^37–40^. Building upon this success, there is a growing interest in applying these techniques to scientific challenges. Specifically, ML methods have shown strong potential to tackle open problems in fluid mechanics^41,42^. ML tools have been further developed for tasks like generation, super-resolution, prediction and inpainting of dynamical systems^43,44^, two-dimensional (2D) and three-dimensional (3D) Eulerian turbulent snapshots^45–50^; see ref. ^51^ for a short summary. In many cases, the validation of these tools when applied to fluid mechanics is primarily limited to simple 2D smooth and quasi-Gaussian turbulent flows or focused on single-point measurements such as mean profiles and two-point spectral properties. There is often a lack of comprehensive quantitative assessments concerning the more intricate multiscale non-Gaussian properties at high Reynolds numbers. Recently, a fully convolutional model has been proposed to generate one-dimensional Eulerian cuts of high-Reynolds-number turbulence^52^. This model has demonstrated success in capturing up to the fourth-order structure function; however, its generalization to higher-order statistics exhibits less accuracy. Given the state of the art, it is fair to say that we lack both equation-informed and data-driven tools to generate 3D single- or multiparticle Lagrangian trajectories possessing statistical and geometrical properties that quantitatively agree with experiments and direct numerical simulations (DNSs). The demand for the synthetic generation of high-quality and high-quantity data is crucial in various turbulent applications, particularly in the Lagrangian domain, where having even a single trajectory requires the reproduction of the entire Eulerian field over huge spatial domains, which is often a daunting or impossible task for DNSs or extremely laborious for experiments.

Here we present a stochastic data-driven model able to match numerical and experimental data concerning single-particle statistics in homogeneous and isotropic turbulence at high Reynolds numbers. The model is based on a state-of-the-art generative DM^36,37,53^. We have trained two distinct DMs for our study: DM-1c, which generates a single component of the Lagrangian velocity, and DM-3c, which simultaneously outputs all three correlated components (Methods). Our synthetic generation protocol is able to reproduce the scaling of velocity increments over the full range of available frequencies and for all statistically converged moments up to the eighth order in the original training data. Moreover, the protocol successfully captures acceleration fluctuations of up to 60 standard deviations and even beyond, including the cross-correlations between the three velocity components. We train the model using high-quality data obtained from DNS at *R*_*λ*_ ≃ 310. The results also show excellent agreement with the numerical ground-truth data for the generalized flatness of fourth, sixth and eighth orders, whose intensities, due to the presence of intermittent fluctuations, are found to be an order of magnitude larger than the expected values in the presence of a Gaussian statistic. Remarkably, our model exhibits strong generalization properties, enabling the synthesis of events with intensities never encountered during the training phase. These extreme fluctuations, resulting from small-scale vortex trapping and sharp u-turn trajectories with unprecedented excursions and rarity, consistently follow the realistic statistics inherent in the training data.

## Problem setup

### Lagrangian turbulence

The dataset used for training is extracted from a high-resolution DNS of the 3D Navier–Stokes equations (NSE) in a cubic periodic domain with large-scale isotropic forcing. Lagrangian point-like particles have an instantaneous velocity, ${{{\bf{V}}}}(t)=\dot{{{{\bf{X}}}}}(t)$, coinciding with the local instantaneous flow streamlines at the particle position, **X**(*t*):

1 $$\dot{{{{\bf{X}}}}}(t)={{{\bf{u}}}}({{{\bf{X}}}}(t),t),$$

where **u** solves the NSE; see equation (6) in Methods. To construct a high-quality ground-truth database, we tracked a total number of trajectories, *N*_p_ = 327,680, each spanning a length of *T* ≃ 1.3*τ*_*L*_ ≃ 200*τ*_*η*_, with a temporal sampling interval of dt_s_ ≃ 0.1*τ*_*η*_. Consequently, each trajectory is discretized into a total of *K* = 2,000 points; see Table 1. Particles are injected randomly in the 3D volume once a statistically stationary evolution is reached for the underlying Eulerian flow, thus ensuring that the Lagrangian statistics are also stationary. The set of multitime observables utilized to benchmark the quality of the single-particle 3D trajectory generation primarily relies on the statistics of Lagrangian velocity increments:

2 $${\delta }_{\tau }{V}_{i}(t)={V}_{i}(t+\tau )-{V}_{i}(t),$$

where *i* = *x*, *y*, *z* indicates any of the three velocity components and *τ* represents the time increment. The instantaneous particle acceleration is obtained from the limit ${a}_{i}(t)=\mathop{\lim }\nolimits_{\tau \to 0}{\delta }_{\tau }{V}_{i}/\tau$, where we use a time resolution of 0.1*τ*_*η*_ for both DNS and DM. The probability density functions (PDFs) of *δ*_*τ*_*V*_*i*_ in Fig. 1a and *a*_*i*_ in Fig. 2 show strongly non-Gaussian fluctuations. The PDFs of *δ*_*τ*_*V*_*i*_ become more pronounced at decreasing the time scale *τ*. It is a well-known empirical fact that Lagrangian velocity increments develop scaling power laws in the inertial range, *τ*_*η*_ ≪ *τ* ≪ *τ*_*L*_, as measured by the Lagrangian structure functions^33,54,55^ of order *p*:

3 $${S}_{\tau }^{(p)}=\langle {({\delta }_{\tau }{V}_{i})}^{p}\rangle \propto {\tau }^{\xi (p)},$$

where with 〈 ⋅ 〉, we indicate an average over all *N*_p_ trajectories and over time. For both DNS and DM-3c, ${S}_{\tau }^{(p)}$ is calculated by further averaging over all velocity components. Henceforth, we neglect the dependence on the velocity component because of isotropy. Concerning the scaling exponents, *ξ*(*p*), there exists a whole spectrum of anomalous corrections, Δ(*p*), to the mean-field dimensional estimate, *p*/2, leading to *ξ*(*p*) = *p*/2 + Δ(*p*). Furthermore, beyond global scaling laws, the statistics of velocity fluctuations can be quantitatively captured scale by scale for each *τ* by measuring the local scaling exponents, which are obtained from the logarithmic derivatives of ${S}_{\tau }^{(p)}$:

4 $$\zeta (p,\tau )=\frac{{\mathrm{d}}\,\log {S}_{\tau }^{(p)}}{{\mathrm{d}}\,\log {S}_{\tau }^{(2)}}.$$

**Table 1:** Eulerian and Lagrangian DNS parameters

*N*_*L*_ 1,024	*L* 2π	dt 1.5 × 10^−4^	*ν* 8 × 10^−4^
*ϵ* 1.8 ± 0.1	*τ*_*η*_ (2.1 ± 0.2) × 10^−2^	*η* (4.2 ± 0.1) × 10^−3^	*R*_*λ*_ ≃310
*N*_p_ 327,680	dt_s_ 2.25 × 10^−3^	*T* 4.5	*K* 2,000

*N*_*L*_ is the resolution in each spatial dimension; *L* is the physical dimension of the cubic periodic box; dt represents the time step in the DNS integration; *ν* stands for kinematic viscosity; *ϵ* = *ν*〈∂_*i*_*u*_*j*_∂_*i*_*u*_*j*_〉 is the total mean energy dissipation, averaged over time and space; ${\tau }_{\eta }=\sqrt{\nu /\epsilon }$ is the Kolmogorov dissipative time; $\eta ={({\nu }^{3}/\epsilon )}^{1/4}$ is the Kolmogorov dissipative scale; *R*_*λ*_ = *u*_rms_*λ*/*ν* signifies the ‘Taylor scale’ Reynolds number, where *u*_rms_ is the root mean squared velocity and $\lambda =\sqrt{5{E}_{\text{tot}}/\Omega }\simeq 0.14$ represents the ‘Taylor scale’, with *E*_tot_ ≃ 4.5 and *Ω* ≃ 1,200 being, respectively, the total mean energy and enstrophy in the flow. Additionally, *τ*_*L*_ = *L*/*u*_rms_≃ 3.5 is the integral time scale. Parameters of the Lagrangian particles are as follows: *N*_p_ is the total number of trajectories, dt_s_ is the time lag between two consecutive Lagrangian dumps, *T* is the total length of each trajectory and *K* = *T*/dt_s_ is the total number of points in each trajectory.

### DMs

**Fig. 3:**
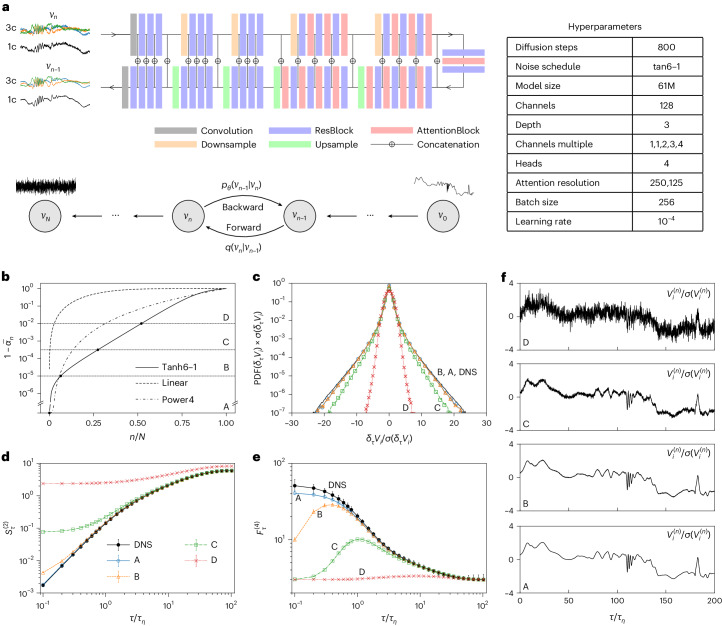
Illustration of the DM and in-depth examination of its backward generation process. **a**, Schematic representation of the DM and associated UNet sketch, complemented by a table of hyperparameters. Here, *N* denotes the total number of diffusion steps and *n* denotes the intermediate step. More details on the network architecture can be found in the Methods section and ref. ^37^. **b**, Three distinct noise schedules for the DM’s forward and backward processes explored in this study (Methods). Points A–D indicate four different stages during the backward generation process (from ${{{{\mathcal{V}}}}}_{N}$ to ${{{{\mathcal{V}}}}}_{0}$) along the optimal noise schedule, curve (tanh6-1). At an early step during the backward process, we have very noisy signals, *n* = 0.52*N* (D), followed by two intermediate steps at *n* = 0.27*N* (C) and *n* = 0.06*N* (B) and the final synthetic trajectory obtained for *n* = 0 (A). **c**–**e**, A few statistical properties of the DM-1c signals generated at the four backward steps A–D: PDF of *δ*_*τ*_*V*_*i*_ for *τ* = *τ*_*η*_ (**c**), second-order structure function, ${S}_{\tau }^{(2)}$ (**d**), fourth-order flatness, ${F}_{\tau }^{(4)}$ (**e**). **f**, Illustration of one trajectory generation from D to A, corresponding to **b**. Source data

DMs emerge in recent years, outperforming the current state-of-the-art GANs on image synthesis^37^. DMs are built upon forward and backward diffusion processes (Fig. 3a and Methods). The forward process is a Markov chain that gradually introduces Gaussian noise into the training data until the original signal is transformed into pure noise. In the opposite direction, the backward process starts from pure Gaussian-noise realizations and learns to progressively denoise the signal, effectively generating the desired data samples, as shown in Fig. 3f. The diffusion processes stem from non-equilibrium statistical physics, leveraging Markov chains to progressively morph one distribution into another^56,57^. The training of DMs involves the use of variational inference lower bound to estimate the loss function along a finite, but large, number of diffusion steps. By focusing on these small incremental changes, the loss term becomes tractable, eliminating the need to resort to the less stable adversarial training, a strategy commonly used by GANs, which aims to reproduce the entire data distribution in a single jump from the input noise. Our implementation of DMs has adopted the UNet architecture of the cutting-edge DM used in computer vision^37^. An optimized noise schedule for the diffusion processes has also been developed to enhance both efficiency and performance when constructing the multiscale features of the signal, as presented in Fig. 3b and discussed in more detail in the Methods.

## Results

### PDFs

In Fig. 1a, we show the success of the DM in generating more and more intense (non-Gaussian) velocity fluctuations, *δ*_*τ*_*V*_*i*_, by sending *τ* → 0, with very good statistical agreement with the ground truth. The typical trajectories generated by DM-1c are also qualitatively shown in Fig. 1b–d for different time lags, *τ*, with local events belonging to both laminar and intense fluctuations. Note the ability of DMs to overcome the additional difficulty of simultaneously generating the three correlated components (DM-3c) required to produce highly complex topological–vortical structures, as shown in Fig. 1e,f. In Fig. 2, we present the PDF of one generic component of the acceleration, *a*_*i*_, from DM-1c, showing very close agreement with the fat-tail ground-truth DNS distribution up to fluctuations around 60–70 times the standard deviation. To illustrate the convergence and generalizability of the DMs, we included results in Figs. 1a and 2 from the DM-1c model trained on only 10% of the DNS data, denoted as DM-1c-10%. The DM-1c and DM-1c-10% results match closely, demonstrating the training convergence. In Fig. 1a, the alignment of DM-1c-10% with the DNS data further underscores the DM’s generalizability to generate extreme events unseen in the training data, which, importantly, adhere to the realistic statistical properties. Further details and comparisons of other statistical measurements for DM-1c-10% are provided in the Supplementary Information.

### Lagrangian structure functions and generalized flatness

**Fig. 4:**
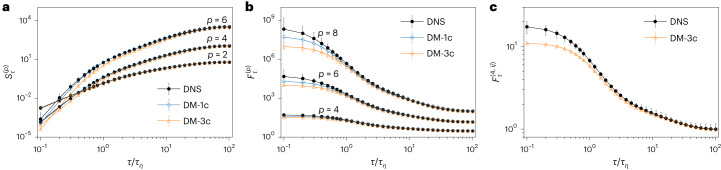
Multiscale statistical properties of velocity increments. **a**, log–log plot of Lagrangian structure functions, ${S}_{\tau }^{(p)}$, for *p* = 2, 4 and 6, compared across DNS, DM-1c and DM-3c. **b**, log–log plot of the generalized flatness, ${F}_{\tau }^{(p)}$, for *p* = 4, 6 and 8, compared across DNS, DM-1c and DM-3c. **c**, log–log plot of fourth-order mixed flatness, ${F}_{\tau }^{(4,ij)}$, averaged over combinations of *ij* = *xy*, *xz* and *yz* for both DNS and DM-3c. The error bars represent the minimum and maximum values obtained for each measure by dividing the entire dataset used to compute the statistics into ten different independent batches of smaller size. Error bars may appear smaller than the data points. Source data

In Fig. 4a, we show for both DM-1c and DM-3c the Lagrangian structure functions given by equation (3) for *p* = 2, 4, 6; and in Fig. 4b, we show the generalized flatness

5 $${F}_{\tau }^{\;(p)}={S}_{\tau }^{(p)}/{[{S}_{\tau }^{(2)}]}^{p/2}.$$

Due to the zero-value odd-order structure functions caused by the symmetry of PDFs of the velocity increments, we focus only on the even orders. Structure functions and generalized flatness of different orders are superimposed with the ground-truth DNS for comparison. The capacity of both DM-1c and DM-3c to reproduce the ground truth over many time-scale decades is striking, especially for *τ* ≳ *τ*_*η*_. However, under the dissipative scale, with *τ* → 0, we observe a tendency for the DM-3c model to generate a slightly smoother signal compared to the DNS, consistent with our observations in Fig. 2. The fourth-order mixed flatness, ${F}_{\tau }^{(4,ij)}=$ $\langle {({\delta }_{\tau }{V}_{i})}^{2}{({\delta }_{\tau }{V}_{j})}^{2}\rangle /{[{S}_{\tau }^{(2)}]}{\,}^{2}$, calculated by averaging over *ij* = *xy*, *xz* and *yz* is shown in Fig. 4c to check the ability of the DM-3c to reproduce the correlation among different components of the velocity vector, confirming quantitatively the agreement between DM-3c and DNS shown in Fig. 1e,f. It is worth noting that although the results are very good, there is still room for further refinement of the scales in the dissipative range.

### Acceleration correlation function

In the inset of Fig. 2, we also present the synthetic single-component acceleration correlation function, *C*_*τ*_ = 〈*a*_*i*_(*t* + *τ*)*a*_*i*_(*t*)〉, where *i* = *x*, *y*, *z*. The result demonstrates a strong alignment with the DNS. This multiscale Lagrangian structure function has been the subject of intense studying and modelling in the past, due to the presence of a whole set of hierarchical time scales affecting its properties^58–61^.

### Local scaling exponents

**Fig. 5:**
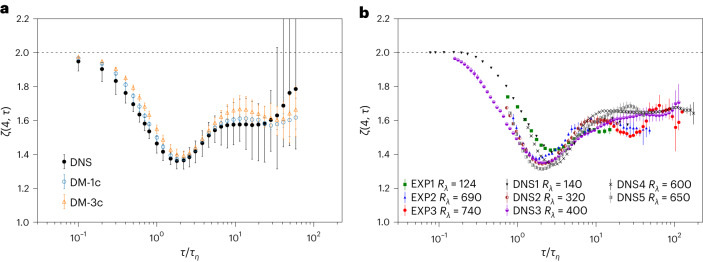
Scale-by-scale intermittent properties. **a**, Comparison between the ground-truth DNS and the two DMs, on the lin-log scale, for the fourth-order logarithmic local slope *ζ*(4, *τ*) defined in equation (4). **b**, The same quantity shown in **a** from a state-of-the-art collection of DNS^84–88^ and experimental data^3,33,89,90^. The dotted horizontal lines represent the non-intermittent dimensional scaling, ${S}_{\tau }^{(4)}\propto {[{S}_{\tau }^{(2)}]}{\,}^{2}$. Statistics and error bars in **a** are derived as in Fig. 4. This resulted in 30 batches for DNS and DM-3c and ten batches for DM-1c. The error bars in panel **b** are computed solely over the three different velocity components. Source data

Let us now introduce what is perhaps the most stringent and quantitative multiscale test for turbulence studies: the comparison of local scaling properties provided by the scale-by-scale exponent defined in equation (4). In practice, we compute *ζ*(*p*, *τ*) by first computing $\text{d}\log {S}_{\tau }^{(p)}/\text{d}\log \tau$ and $\text{d}\log {S}_{\tau }^{(2)}/\text{d}\log \tau$ on a grid with *τ* intervals of 1 (from 1 to 1,024) using second-order accurate central differences and then performing the division. It is easy to realize that in the inertial range, where equation (3) is supposed to hold, we have *ζ*(*p*, *τ*) = *ξ*(*p*)/*ξ*(2), independently of *τ*. On the other hand, it is known that most of the ‘turbulent’ deadlocks develop at the interface between viscous and inertial ranges, *τ* ≈ *τ*_*η*_, where the highest level of non-Gaussian fluctuations is observed. Multifractal statistical models are able to fit the whole complexity of the *ζ*(*p*, *τ*) curves in the entire range of time scales^33,54,62,63^. This is achieved by introducing a multiplicative cascade model in the inertial range, ended with a fluctuating dissipative time scale, ${\tilde{\tau }}_{\eta }$ (refs. ^64,65^). Despite numerous attempts, we miss a proper constructive method for embedding the above phenomenology to generate synthetic, realistic 3D Lagrangian trajectories^27,29,32,66^. In Fig. 5a, we show the local exponent for *p* = 4 for DM-1c and DM-3c and for the DNS data used for training. For comparison, in Fig. 5b we show a state-of-the-art collection of experimental and other DNS data published in the past. Similar results are obtained for *p* = 6 and 8 (not shown). The agreement of results from DMs with experimental and DNS data is remarkable. This is considered a high-quality benchmark, demanding the reproduction of the rate of variation of the local scaling properties over a range of frequencies/time lags spanning more than three decades and a corresponding variation of the structure functions (equation (3)) over four to five decades (Fig. 4). Such substantial variations are distilled into the measurement of *O*(1) quantities (equation (4)) with an error margin within 5%. There are no other tests that can check the scaling properties with greater precision because statistical accuracy typically does not allow one to go beyond a simple—and inaccurate—log–log fit of scaling laws over the full range of variation.

## Discussion

**Fig. 6:**
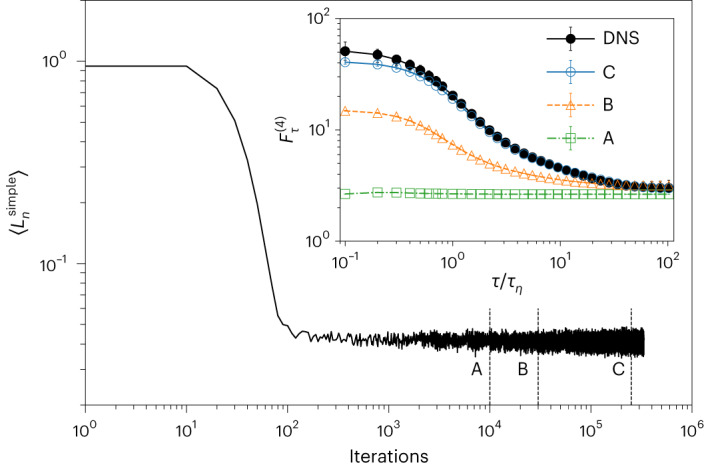
DM training protocol. The training loss function, $\langle {L}_{n}^{{{{\rm{simple}}}}}\rangle$, against iterations for DM-1c. Here, 〈 ⋅ 〉 represents the average over a batch of training data, each of which has a corresponding random step *n* with 0 ≤ *n* ≤ *N*. The inset presents the fourth-order flatness obtained from DM-1c at different iterations (A: 10 × 10^3^, B: 30 × 10^3^ C: 250 × 10^3^), in comparison with that from DNS data. Statistics and error bars are derived as in Fig. 4. Source data

We have presented a data-driven model capable of reproducing all recognized statistical properties of single-particle Lagrangian turbulence in homogeneous and isotropic turbulence from the large scales down to the inertial and inertial-viscous scaling range, including the enhanced intermittent properties observed around *τ*_*η*_. This achievement is summarized by the PDFs of velocity increments in the inertial range and acceleration (Figs. 1 and 2) as well as by the structure functions, the flatness among different components and the local scaling exponents as shown in Figs. 4 and 5. In Table 2, we further summarize a comparison of single-time two-point correlations of velocity and acceleration, showing excellent matching of DM synthetic data with DNS, except for the case of cross correlation among different acceleration components, Σ^*A*^, where DM-3c gives a smaller value than DNS. This trend is also reflected in the smoother transition observed in the limit *τ* → 0 for the single- and mixed-component flatness in Fig. 4b,c. Furthermore, it is important to highlight the ability of both DM-1c and DM-3c to break the deadlock of viscous intermittency by being able to reproduce the dip structure in the local scaling exponents, as shown in Fig. 5 in the range *τ* ≈ *τ*_*η*_. Fig. 6 shows how DM generation improves multiscale statistics as training progresses. We also evaluated another prominent generative model, the Wasserstein GAN, for this task. Despite efforts to train and select the best-performing model, its accuracy was only satisfactory at large and intermediate scales and failed considerably at smaller time scales. Further details can be found in the Supplementary Information.

**Table 2:** Single-time second-order correlations

	DNS	DM-1c	DM-3c
*E*	3.0	3.0	2.9
*A*	1.7 × 10^−3^	1.8 × 10^−3^	1.6 × 10^−3^
Σ^*V*^	− 0.41	${{\emptyset}}$	− 0.39
Σ^*A*^	4.4 × 10^−5^	${{\emptyset}}$	2.4 × 10^−5^

Quantities are related to both velocity and acceleration for DNS, DM-1c and DM-3c: $E=1/3{\sum }_{i}\langle {V}_{i}^{2}\rangle ,A=1/3{\sum }_{i}\langle {a}_{i}^{2}\rangle ,{\Sigma }^{V}=1/3{\sum }_{i,j}\langle {V}_{i}^{2}{V}_{j}^{2}\rangle -\langle {V}_{i}^{2}\rangle \langle {V}_{j}^{2}\rangle ,{\Sigma }^{A}$ $=1/3{\sum }_{i,j}\langle {a}_{i}^{2}{a}_{j}^{2}\rangle -\langle {a}_{i}^{2}\rangle \langle {a}_{j}^{2}\rangle$, where in the last two expressions, the summation is only for *ij* = *xy*, *xz* and *yz*.

### Generalizability

Having AI models capable of generating high-quality trajectories can considerably increase the availability of well-validated synthetic data for pretraining physical applications based on Lagrangian single-particle dispersion. Even more surprisingly, our DM shows the ability to generate trajectories with extremely intense events, thus generalizing beyond the information absorbed during the training phase while still preserving realistic statistical properties. This is clearly illustrated by the striking observation of the extended tails of the PDFs measured from the larger dataset generated by the DM compared to those measured from the smaller set of training data, as shown in Figs. 1a and 2. Currently, our DM is not configured to generalize to different flow configurations, such as different boundary conditions, forcing mechanisms or higher Reynolds numbers. Achieving this adaptability may require the use of a conditional diffusion model^37,53^. By integrating data composed of diverse flows and geometries, such a model could interpolate between different setups and adapt to new conditions, providing a promising avenue for future research.

### Explainability

The fundamental physical model learned by the DM to generate the correct set of multitime fluctuations remains elusive. The DM is based on nested non-linear Gaussian denoising, resembling in spirit the multiscale buildup of fluctuations used in the creation of multifractal signals and measures. The progressive enrichment of signal properties along the backward diffusion process is displayed in Fig. 3c–f. In Fig. 3e, we show quantitatively the buildup of non-trivial flatness at different stages of the backward process. Similarly, but more qualitatively, Fig. 3f shows the emerging non-Gaussian and non-trivial properties within a single trajectory, transitioning from a very noisy signal (*n* = 0.52*N*) to the final step of the backward process (*n* = 0). Figure 3c–f illustrates that during the generation process, the model initially generates statistics at larger scales and gradually builds up statistics at smaller scales. Decrypting this multiscale process in terms of precise non-linear mapping could lead to important discoveries in our phenomenological understanding of turbulence. A promising approach to enhance the interpretability of the model is to factorize the data with wavelet decomposition and implement DMs to synthesize the wavelet coefficients, conditioning on the low-frequency ones^67^.

### Impact

Synthetic stochastic generative models offer remarkable advantages. They (1) provide access to open data without copyright or ethical issues connected to real-data usage and (2) enable the production of high-quality and high-quantity datasets, which can be used to train other models that require such data as input. The ultimate goal is to provide synthetic datasets that enable new models for downstream applications to reach enhanced accuracy, replacing the necessity for real-data pretraining with synthetic pretraining. Our study opens the way for addressing many questions for which the use of real Lagrangian trajectories requires an unfeasible computational or experimental effort. These questions include the relative dispersion problem between two or more particles to study Richardson diffusion^68,69^, shape dynamics^70,71^, data augmentation of datasets for drifter trajectories in specific oceanic applications^72,73^, generation and classification of inertial particle trajectories^8^ and data inpainting^48^.

## Methods

### Navier–Stokes simulations for Lagrangian tracers

We solve the 3D NSE:

6 $$\left\{\begin{array}{l}{\partial }_{t}{{{{{\mathbf{u}}}}}}+{{{{{\mathbf{u}}}}}}\cdot \nabla {{{{{\mathbf{u}}}}}}=-\nabla p+\nu \Delta {{{{{\mathbf{u}}}}}}+{{{\bf{F}}}}\quad \\ \nabla \cdot {{{{{\mathbf{u}}}}}}=0\quad \end{array}\right.,$$

for an incompressible fluid of viscosity *ν*^17^. The flow is driven to a non-equilibrium statistically steady state by a homogeneous and isotropic forcing, **F**, obtained via a second-order Ornstein–Uhlenbeck process^18^. For the DNS of the Eulerian field, we used a standard pseudospectral solver fully dealiased with the two-thirds rule. Details on the simulation can be found in ref. ^74^. Parameters of the DNS used in this work are given in Table 1. The database of Lagrangian trajectories used in this study is dumped each dt_s_ = 15dt ≃ 0.1*τ*_*η*_ (ref. ^75^). Lagrangian integration of tracers is obtained via a B-spline sixth-order interpolation scheme to obtain the fluid velocity at the particle position and with a second-order Adams–Bashforth time-marching scheme^76^.

### DMs

The specific implementation of DMs utilized in this work is based on recent research^37^ that demonstrated extremely good performances of DMs even in comparison with GAN for image synthesis. The network architecture, depicted in Fig. 3, relies on the typical UNet structure^77^, which is commonly used for image analysis tasks as it is designed to capture both high-level contextual information and precise spatial detail. The UNet consists of two primary components: a contracting and an expanding path. Acting as an encoder, the contracting path progressively reduces the spatial dimension of the input data while increasingly extracting abstract features that contain the global context of the input data. The expanding path acts as a decoder, interpreting the learned features and systematically recovering the spatial resolution to generate the final output (see the later section ‘DM architecture and noise schedule’ and Fig. 3 for more details).

### Training algorithm

We train two different classes of DM: one to generate a single component of the Lagrangian velocity field (DM-1c) and one for the three components simultaneously (DM-3c). Let us denote each entire trajectory as ${{{\mathcal{V}}}}$, where

$${{{\mathcal{V}}}}=\{{V}_{i}({t}_{k})| {t}_{k}\in [0,T];i=x,y,z\};\qquad \,{{\mbox{(DM-1c)}}}\,$$

and

$${{{\mathcal{V}}}}=\{{V}_{x}({t}_{k}),{V}_{y}({t}_{k}),{V}_{z}({t}_{k})\,| {t}_{k}\in [0,T]\};\qquad \,{{\mbox{(DM-3c)}}}\,$$

and *k* = 1, …, *K* goes over the total number of discretized sampling times for each trajectory (Table 1). The distribution of the ground-truth trajectories obtained from DNS of the NSE is denoted as $q({{{\mathcal{V}}}})$. We introduce a forward noising process that starts from the ground-truth trajectory ${{{{\mathcal{V}}}}}_{0}={{{\mathcal{V}}}}$ and transforms it, after *N* steps, to a set of trajectories identical to pure random uncorrelated Gaussian noise. This process generates latent variables ${{{{\mathcal{V}}}}}_{1},\ldots ,{{{{\mathcal{V}}}}}_{N}$ by introducing Gaussian noise at step *n* with a variance *β*_*n*_ ∈ (0, 1) according to the following formulation

7 $$q({{{{\mathcal{V}}}}}_{1:N}| {{{{\mathcal{V}}}}}_{0}):=\mathop{\prod }\limits_{n=1}^{N}q({{{{\mathcal{V}}}}}_{n}| {{{{\mathcal{V}}}}}_{n-1}),$$

where we have introduced the shorthand notation ${{{{\mathcal{V}}}}}_{1:N}$ to denote the entire chain of the ensemble of noisy trajectories ${{{{\mathcal{V}}}}}_{1},{{{{\mathcal{V}}}}}_{2},\ldots ,{{{{\mathcal{V}}}}}_{N}$, and given that the tilde represents ‘distributed as’, each step is defined as

8 $$q({{{{\mathcal{V}}}}}_{n}| {{{{\mathcal{V}}}}}_{n-1})\to {{{{\mathcal{V}}}}}_{n} \sim {{{\mathcal{N}}}}\left(\sqrt{1-{\beta }_{n}}{{{{\mathcal{V}}}}}_{n-1},{\beta }_{n}{{{\bf{I}}}}\right).$$

Equation (7) is obtained using the Markovian property of the *n* steps in the forward process. For a large enough *N* and a suitable sequence of *β*_*n*_, the latent vector ${{{{\mathcal{V}}}}}_{N} \sim {{{\mathcal{N}}}}(0,{{{\bf{I}}}})$ approximates a delta-correlated Gaussian signal with zero mean and unitary variance. A second remarkable property of the above process, which follows from the Gaussian property of the noise introduced at each step (equation (8)), is that given ${{{{\mathcal{V}}}}}_{0}$, we can sample ${{{{\mathcal{V}}}}}_{n}$ at any given arbitrary *n* in a closed form by defining *α*_*n*_: = 1 − *β*_*n*_ and ${\bar{\alpha }}_{n}:=\mathop{\prod }\nolimits_{i = 0}^{n}{\alpha }_{i}$ as

9 $$q({{{{\mathcal{V}}}}}_{n}| {{{{\mathcal{V}}}}}_{0})\to {{{{\mathcal{V}}}}}_{n} \sim {{{\mathcal{N}}}}(\sqrt{{\bar{\alpha }}_{n}}{{{{\mathcal{V}}}}}_{0},(1-{\bar{\alpha }}_{n}){{{\bf{I}}}}).$$

In other words, starting from any ground-truth trajectory, ${{{{\mathcal{V}}}}}_{0}$, we can evaluate its corresponding realization after *n* steps in the forward process as

10 $${{{{\mathcal{V}}}}}_{n}=\sqrt{{\bar{\alpha }}_{n}}{{{{\mathcal{V}}}}}_{0}+\sqrt{1-{\bar{\alpha }}_{n}}\epsilon ,$$

where $\epsilon \sim {{{\mathcal{N}}}}({{{\boldsymbol{0}}}},{{{\bf{I}}}})$. Now, it is clear that if we can reverse the above process and sample from $p({{{{\mathcal{V}}}}}_{n-1}| {{{{\mathcal{V}}}}}_{n})$, we will be able to generate new true samples starting from the Gaussian-noise input, $p({{{{\mathcal{V}}}}}_{N})={{{\mathcal{N}}}}({{{\boldsymbol{0}}}},{{{\bf{I}}}})$. In general, the backward distribution, $p({{{{\mathcal{V}}}}}_{n-1}| {{{{\mathcal{V}}}}}_{n})$, is unknown. However, in the limit of continuous diffusion (small *β*_*n*_), the reverse process has the identical functional form of the forward process^56^. Because $q({{{{\mathcal{V}}}}}_{n}| {{{{\mathcal{V}}}}}_{n-1})$ is a Gaussian distribution and *β*_*n*_ is chosen to be small, $p({{{{\mathcal{V}}}}}_{n-1}| {{{{\mathcal{V}}}}}_{n})$ will also be a Gaussian. In this way, the UNet, with trainable parameters *θ*, needs to model the mean ${\mu }_{\theta }({{{{\mathcal{V}}}}}_{n},n)$ and standard deviation ${\Sigma }_{\theta }({{{{\mathcal{V}}}}}_{n},n)$ of the transition probabilities for all steps in the backward diffusion process:

11 $${p}_{\theta }({{{{\mathcal{V}}}}}_{0:N})=p({{{{\mathcal{V}}}}}_{N})\mathop{\prod }\limits_{n=1}^{N}{p}_{\theta }({{{{\mathcal{V}}}}}_{n-1}| {{{{\mathcal{V}}}}}_{n}),$$

where each reverse step can be written as

12 $${p}_{\theta }({{{{\mathcal{V}}}}}_{n-1}| {{{{\mathcal{V}}}}}_{n})\to {{{{\mathcal{V}}}}}_{n-1} \sim {{{\mathcal{N}}}}({\mu }_{\theta }({{{{\mathcal{V}}}}}_{n},n),{\Sigma }_{\theta }({{{{\mathcal{V}}}}}_{n},n)).$$

During training, the optimization involves minimizing the cross entropy, *L*_CE_, between the ground-truth distribution and the likelihood of the generated data

13 $$\begin{array}{rcl}{L}_{\text{CE}}&:=&-{{\mathbb{E}}}_{q({{{{\mathcal{V}}}}}_{0})}\log \left({p}_{\theta }({{{{\mathcal{V}}}}}_{0})\right)\\ &=&-{{\mathbb{E}}}_{q({{{{\mathcal{V}}}}}_{0})}\log \left(\displaystyle\int{p}_{\theta }({{{{\mathcal{V}}}}}_{0:N})d{{{{\mathcal{V}}}}}_{1:N}\right).\end{array}$$

However, integrating over all possible backward paths from 1 to *N* and averaging over all ground-truth data, ${{\mathbb{E}}}_{q({{{{\mathcal{V}}}}}_{0})}[\ldots ]=\int[\ldots ]q({{{{\mathcal{V}}}}}_{0})d{{{{\mathcal{V}}}}}_{0}$, to evaluate every network update is beyond being numerically intractable. A way out is to exploit a variational lower bound *L*_VLB_ for the cross entropy^56^:

14 $$\begin{array}{rc}&{L}_{\text{CE}}\le {{\mathbb{E}}}_{q({{{{\mathcal{V}}}}}_{0})}{{\mathbb{E}}}_{p({{{{\mathcal{V}}}}}_{1:N}| {{{{\mathcal{V}}}}}_{0})}\left[\log \frac{p({{{{\mathcal{V}}}}}_{1:N}| {{{{\mathcal{V}}}}}_{0})}{{p}_{\theta }({{{{\mathcal{V}}}}}_{0:N})}\right]:={L}_{\text{VLB}}.\end{array}$$

To make the above expression computable, the expectation value can be split into its independent steps. Consequently, it can be rewritten as a summation of several Kullback–Leibler divergences, *D*_KL_, plus one entropy term (see details in Appendix B of ref. ^56^). In this way, *L*_VLB_ becomes

15 $$\begin{array}{rcl}{L}_{\text{VLB}}&=&{{\mathbb{E}}}_{q({{{{\mathcal{V}}}}}_{0})}\left[\underbrace{{D}_{{{{\rm{KL}}}}}\!\left(p({{{{\mathcal{V}}}}}_{N}| {{{{\mathcal{V}}}}}_{0})\parallel {p}_{\theta }({{{{\mathcal{V}}}}}_{N})\right)}_{\begin{array}{c}{L}_{N}\end{array}}\right.\\ &&+\mathop{\sum }\limits_{n > 1}^{N}\underbrace{{D}_{{{{\rm{KL}}}}}\!\left(p({{{{\mathcal{V}}}}}_{n-1}| {{{{\mathcal{V}}}}}_{n},{{{{\mathcal{V}}}}}_{0})\parallel {p}_{\theta }({{{{\mathcal{V}}}}}_{n-1}| {{{{\mathcal{V}}}}}_{n})\right)}_{\begin{array}{c}{L}_{n-1}\end{array}}\\ &&\left.\underbrace{-\log {p}_{\theta }({{{{\mathcal{V}}}}_{0}| {{{{\mathcal{V}}}}}_{1})}}_{\begin{array}{c}{L}_{0}\end{array}}\right].\end{array}$$

The first term, *L*_*N*_, can be ignored during training, as $p({{{{\mathcal{V}}}}}_{N}| {{{{\mathcal{V}}}}}_{0})$ does not depend on the network hyperparameters, and ${p}_{\theta }({{{{\mathcal{V}}}}}_{N})={{{\mathcal{N}}}}(0,{{{\bf{I}}}})$ is just the Gaussian distribution. Hence, the network must minimize only the terms *L*_*n*_ with *n* < *N* to reproduce the entire backward diffusion process and generate correct data. At this point, the last remarkable property that allows each term of the variational lower bound to be written in a tractable way is that the inverse conditional probability can be calculated analytically when conditioned on a particular realization of the ground-truth data. Using Bayes’ theorem, we can write

16 $$p({{{{\mathcal{V}}}}}_{n-1}| {{{{\mathcal{V}}}}}_{n},{{{{\mathcal{V}}}}}_{0})=q({{{{\mathcal{V}}}}}_{n}| {{{{\mathcal{V}}}}}_{n-1},{{{{\mathcal{V}}}}}_{0})\frac{q({{{{\mathcal{V}}}}}_{n-1}| {{{{\mathcal{V}}}}}_{0})}{q({{{{\mathcal{V}}}}}_{n}| {{{{\mathcal{V}}}}}_{0})}.$$

All probabilities in the right-hand side of equation (16) describe forward steps as defined in equations (8) and (9). Therefore, equation (16) can be regarded as the product of three Gaussians

17 $$\begin{array}{rcl}p({{{{\mathcal{V}}}}}_{n-1}| {{{{\mathcal{V}}}}}_{n},{{{{\mathcal{V}}}}}_{0})&\propto &\exp \left(-\frac{{({{{{\mathcal{V}}}}}_{n}-\sqrt{{\alpha }_{n}}{{{{\mathcal{V}}}}}_{n-1})}^{2}}{2{\beta }_{n}}\right)\\ &&\cdot \exp \left(-\frac{{({{{{\mathcal{V}}}}}_{n-1}-\sqrt{{\bar{\alpha }}_{n-1}}{{{{\mathcal{V}}}}}_{0})}^{2}}{2(1-{\bar{\alpha }}_{n-1})}\right)\\ &&\cdot \exp \left(\frac{{({{{{\mathcal{V}}}}}_{n}-\sqrt{{\bar{\alpha }}_{n}}{{{{\mathcal{V}}}}}_{0})}^{2}}{2(1-{\bar{\alpha }}_{n})}\right),\end{array}$$

which can be rewritten as

18 $$p({{{{\mathcal{V}}}}}_{n-1}| {{{{\mathcal{V}}}}}_{n},{{{{\mathcal{V}}}}}_{0})\to {{{{\mathcal{V}}}}}_{n-1} \sim {{{\mathcal{N}}}}(\tilde{\mu }({{{{\mathcal{V}}}}}_{n},{{{{\mathcal{V}}}}}_{0}),{\tilde{\beta }}_{n}{{{\bf{I}}}}),$$

where the mean and the standard deviation of the conditioned reverse probability are, respectively,

19 $${\tilde{\mu }}_{n}({{{{\mathcal{V}}}}}_{n},{{{{\mathcal{V}}}}}_{0}):=\frac{\sqrt{{\bar{\alpha }}_{n-1}}{\beta }_{n}}{1-{\bar{\alpha }}_{n}}{{{{\mathcal{V}}}}}_{0}+\frac{\sqrt{{\alpha }_{n}}(1-{\bar{\alpha }}_{n-1})}{1-{\bar{\alpha }}_{n}}{{{{\mathcal{V}}}}}_{n}$$

and

20 $${\tilde{\beta }}_{n}:=\frac{1-{\bar{\alpha }}_{n-1}}{1-{\bar{\alpha }}_{n}}{\beta }_{n}.$$

All terms denoted by *L*_*n*−1_ in the variational lower bound are *D*_KL_ between the two Gaussians that depend only on the difference between their mean values and standard deviations. Assuming that the standard deviations of the reverse and forward processes are identical, that is, Σ_*θ*_ = *β*_*n*_**I**, we only need to model the mean values of the backward Gaussians. Consequently, the Kullback–Leibler divergence simplifies to the difference between the two mean values, given in equation (19) and the output of the UNet mode, ${\mu }_{\theta }({{{{\mathcal{V}}}}}_{n},n)$, in equation (12). From this simplification, it follows that each loss term becomes

$${L}_{n-1}={{\mathbb{E}}}_{q({{{{\mathcal{V}}}}}_{0})}\left[\frac{1}{2{\beta }_{n}}| | {\tilde{\mu }}_{n}({{{{\mathcal{V}}}}}_{n},{{{{\mathcal{V}}}}}_{0})-{\mu }_{\theta }({{{{\mathcal{V}}}}}_{n},n)| {| }^{2}\right].$$

Expressing ${{{{\mathcal{V}}}}}_{0}$ in term of ${{{{\mathcal{V}}}}}_{n}$ by inverting equation (10) and substituting it in equation (19), the mean value of the reverse conditioned probability can be rewritten as

21 $$\tilde{\mu }({{{{\mathcal{V}}}}}_{n},{{{{\mathcal{V}}}}}_{0})=\frac{1}{\sqrt{{\alpha }_{n}}}\left({{{{\mathcal{V}}}}}_{n}-\frac{{\beta }_{n}}{\sqrt{1-{\bar{\alpha }}_{n}}}{\boldsymbol{\epsilon }}_{{{{{\mathcal{V}}}}}_{0},n}\right),$$

where the subscripts of the noise term, ${{{{\boldsymbol{\epsilon }}}}}_{{{{{\mathcal{V}}}}}_{0},n}$, indicate that this is the specific noise realization used to obtain ${{{{\mathcal{V}}}}}_{n}$ from ${{{{\mathcal{V}}}}}_{0}$, as defined in equation (10). Now, because ${{{{\mathcal{V}}}}}_{n}$ is known by the network, one may reparameterize the predicted mean ${\mu }_{\theta }({{{{\mathcal{V}}}}}_{n},n)$ as

22 $${\mu }_{\theta }({{{{\mathcal{V}}}}}_{n},n)=\frac{1}{\sqrt{{\alpha }_{n}}}\left({{{{\mathcal{V}}}}}_{n}-\frac{{\beta }_{n}}{\sqrt{1-{\bar{\alpha }}_{n}}}{{{{\boldsymbol{\epsilon }}}}}_{\theta }({{{{\mathcal{V}}}}}_{n},n)\right),$$

where ***ϵ***_*θ*_ is a function approximator designed to predict ${{{{\boldsymbol{\epsilon }}}}}_{{{{{\mathcal{V}}}}}_{0},n}$ from ${{{{\mathcal{V}}}}}_{n}$, leading to the following reformulation of the loss terms:

$${L}_{n-1}={{\mathbb{E}}}_{q({{{{\mathcal{V}}}}}_{0}),{{{{\boldsymbol{\epsilon }}}}}_{{}_{{{{{\mathcal{V}}}}}_{0},n}}}\left[\frac{{\beta }_{n}}{2{\alpha }_{n}(1-{\bar{\alpha }}_{n})}| | {{{{\boldsymbol{\epsilon }}}}}_{{{{{\mathcal{V}}}}}_{0},n}-{{{{\boldsymbol{\epsilon }}}}}_{\theta }({{{{\mathcal{V}}}}}_{n},n)| {| }^{2}\right].$$

Namely, in the training *ϵ*_*θ*_ predicted from the DM is compared with the one used to build up the ${{{{\mathcal{V}}}}}_{n}$ from ${{{{\mathcal{V}}}}}_{0}$. This formulation leads to faster and more stable training^36^. Moreover, it has been shown^36^ that one can obtain good results even by performing the training without learning the variance of the reverse process and introducing a simpler, reweighted loss function defined as

23 $${L}_{n-1}^{{{{\rm{simple}}}}}={{\mathbb{E}}}_{q({{{{\mathcal{V}}}}}_{0}),{{{{\boldsymbol{\epsilon }}}}}_{{{{{\mathcal{V}}}}_{0},n}}}\left[| | {{{{\boldsymbol{\epsilon }}}}}_{{{{{\mathcal{V}}}}}_{0},n}-{{{{\boldsymbol{\epsilon }}}}}_{\theta }({{{{\mathcal{V}}}}}_{n},n)| {| }^{2}\right],$$

which is identical to the one we implemented in this work. It is worth noting that due to the Gaussian form of ${p}_{\theta }({{{{\mathcal{V}}}}}_{0}| {{{{\mathcal{V}}}}}_{1}),{L}_{0}$ results in the same loss function as depicted in equation (23). Therefore, the optimized loss functions can be expressed as ${L}_{n}^{{{{\rm{simple}}}}}$, where *n* ranges from 0 to *N* − 1.

### DM architecture and noise schedule

The UNet architecture we have implemented is one of the most advanced networks described in the literature, demonstrating state-of-the-art performance in image generation^37^. It is capable of extracting hidden, spatially correlated information that is essential both for image generation and for accomplishing our specific task. The details of the architecture, including the hyperparameters, are summarized in the table in Fig. 3a. Each encoder and decoder part consists of five levels. Progressing to the next level entails doubling or halving the resolution as one passes through an Upsample or Downsample layer, respectively. The Depth parameter controls the number of ResBlocks with or without AttentionBlocks at each level. Within each level, layers share the same number of features, which can be determined using the Channels and Channels multiple parameters from the table. Attention mechanisms^78^ allow neural networks to prioritize certain regions or features within the data. In this study, we employed multihead attention with four heads. AttentionBlocks were utilized at levels with resolutions of 250 and 125. For the DM-1c model, we utilized 250 × 10^3^ iterations, while 400 × 10^3^ iterations were used for the DM-3c model. In each iteration, we sample a batch of training data and assign a random step index *n* to each sample and then optimize ${L}_{n}^{{{{\rm{simple}}}}}$ across the data batch. Figure 6 shows the training loss as a function of iteration for DM-1c alongside the fourth-order flatness of samples generated from it at different iteration checkpoints: A, B and C. Here, C corresponds to the final model. It reveals that although the loss rapidly reached a ‘plateau’, it is crucial to continue training for the model convergence. This is because $\langle {L}_{n}^{{{{\rm{simple}}}}}\rangle$ is an average derived from a data batch where each sample is assigned a random *n*, which does not truly represent the inherent loss *L*_CE_ described in equation (13). Although *L*_CE_ can be approximated as the summed expectation of ${L}_{n}^{{{{\rm{simple}}}}}$ across the training dataset for 0 < *n* ≤ *N*, direct evaluation of *L*_CE_ is impractical. Instead, we rely on examining the statistical properties to measure training progress.

Concerning the noise schedule to improve the training and sampling protocols, we explored three different laws and found that the optimal one for our application is given in terms of a tanh profile; see Fig. 3b. Indeed, all results shown in the main text and in panels Fig. 3c–e of the same figure have been obtained by following the schedule (tanh6-1):

24 $${\bar{\alpha }}_{n}=\frac{-\tanh (7n/N-6)+\tanh 1}{-\tanh (-6)+\tanh 1},$$

which allowed us to use *N* = 800 diffusion steps rather than *N* = 4,000 needed for the linear case where the forward process variances are constantly increasing from *β*_1_ = 10^−4^ to *β*_*N*_ = 0.02. As a result, a fivefold improvement in performance is achieved. We also explored an alternative noise schedule (power4) with a functional form: ${\bar{\alpha }}_{n}=1-{(n/N)}^{4}$, with *N* = 800, which resulted in being slightly inferior to (tanh6-1). Note that applying methods to speed up DM sampling with pretrained models remains worthy of future exploration^79,80^.

### Computational cost

To illustrate the computational cost of our case, the DNS of the Eulerian field takes about 7.2 hours on 4,096 cores. This step is essential even to generate a single Lagrangian trajectory. An additional 64% of the time is required to track 4 million Lagrangian tracers. All training and sampling of the DMs in our study was performed on four NVIDIA A100 GPUs. Training takes approximately 1 hour per 10,000 iterations, resulting in approximately 25 hours for DM-1c and 40 hours for DM-3c. Sampling an equivalent number of 4 million trajectories takes about 200 hours.

## Supplementary information

Supplementary Information Supplementary Figs. 1 and 2 and Discussion.

Supplementary Data Source data for Supplementary Fig. 1.

Supplementary Data Source data for Supplementary Fig. 2.

## Source data

Source Data Fig. 1 Source data for Fig. 1.

Source Data Fig. 2 Source data for Fig. 2.

Source Data Fig. 3 Source data for Fig. 3.

Source Data Fig. 4 Source data for Fig. 4.

Source Data Fig. 5 Source data for Fig. 5.

Source Data Fig. 6 Source data for Fig. 6.

## Data Availability

The Lagrangian trajectories used in this study, which include the positions, velocities and accelerations of each particle, are available for download from the open access Smart-TURB portal http://smart-turb.roma2.infn.it, in the TURB-Lagr repository^74,75^. It is also possible to download from the same repository a minimum dataset for testing the code and the generated Lagrangian trajectories (velocities over time) used for all analyses in this paper. TURB-Lagr is a database of 3D turbulent Lagrangian trajectories obtained by DNS of the NSE with homogeneous and isotropic forcing. Details on how to download and read the database are also given in the portal. All data related to this study have also been uploaded to the Open Access Repository (10.15161/oar.it/143615)^81^. Source data are provided with this paper.

## Code availability

The code to train the DM and generate new trajectories can be found at https://github.com/SmartTURB/diffusion-lagr (ref. ^82^). A ready-to-run Code Ocean capsule with the complete environment is available at https://codeocean.com/capsule/0870187/tree/v1 (ref. ^83^).

## References

[1] Shraiman, I. B. & D. Siggia, D. E. Scalar turbulence. *Nature* **405**, 639–646 (2000). 10864314 10.1038/35015000

[2] La Porta, A., Voth, G. A., Crawford, A. M., Alexander, J. & Bodenschatz, E. Fluid particle accelerations in fully developed turbulence. *Nature* **409**, 1017–1019 (2001). 11234005 10.1038/35059027

[3] Mordant, N., Metz, P., Michel, O. & Pinton, J.-F. Measurement of lagrangian velocity in fully developed turbulence. *Phys. Rev. Lett.* **87**, 214501 (2001). 11736341 10.1103/PhysRevLett.87.214501

[4] Falkovich, G., Gawȩdzki, K. & Vergassola, M. Particles and fields in fluid turbulence. *Rev. Mod. Phys.* **73**, 913–975 (2001).

[5] Yeung, P. Lagrangian investigations of turbulence. *Annu. Rev. Fluid Mech.* **34**, 115–142 (2002).

[6] Pomeau, Y. The long and winding road. *Nat. Phys.* **12**, 198–199 (2016).

[7] Falkovich, G. & Sreenivasan, K. R. Lessons from hydrodynamic turbulence. *Phys. Today* **59**, 43 (2006).

[8] Toschi, F. & Bodenschatz, E. Lagrangian properties of particles in turbulence. *Annu. Rev. fluid Mech.* **41**, 375–404 (2009).

[9] Shaw, R. A. Particle-turbulence interactions in atmospheric clouds. *Annu. Rev. Fluid Mech.* **35**, 183–227 (2003).

[10] McKee, C. F. & Stone, J. M. Turbulence in the heavens. *Nat. Astron.* **5**, 342–343 (2021).

[11] Bentkamp, L., Lalescu, C. C. & Wilczek, M. Persistent accelerations disentangle lagrangian turbulence. *Nat. Commun.* **10**, 3550 (2019). 31391458 10.1038/s41467-019-11060-9 PMC6685982

[12] Sawford, B. L. & Pinton, J.-F. in *Ten Chapters in Turbulance* (eds. Davidson, P. A., Kaneda, Y. & Sreenivasan, K. R.) 132–175 (Cambridge Univ. Press, 2013).

[13] Xia, H., Francois, N., Punzmann, H. & Shats, M. Lagrangian scale of particle dispersion in turbulence. *Nat. Commun.* **4**, 2013 (2013). 23771051 10.1038/ncomms3013

[14] Barenghi, C. F., Skrbek, L. & Sreenivasan, K. R. Introduction to quantum turbulence. *Proc. Natl Acad. Sci. USA* **111**, 4647–4652 (2014). 24704870 10.1073/pnas.1400033111 PMC3970860

[15] Xu, H. et al. Flight–crash events in turbulence. *Proc. Natl Acad. Sci. USA* **111**, 7558–7563 (2014). 24794529 10.1073/pnas.1321682111 PMC4040622

[16] Laussy, F. P. Shining light on turbulence. *Nat. Photonics* **17**, 381–382 (2023).

[17] Frisch, U.*Turbulence: The Legacy of AN Kolmogorov* (Cambridge Univ. Press, 1995).

[18] Sawford, B. L. Reynolds number effects in Lagrangian stochastic models of turbulent dispersion. *Phys. Fluids A* **3**, 1577–1586 (1991).

[19] Pope, S. B. Simple models of turbulent flows. *Phys. Fluids* **23**, 011301 (2011).

[20] Viggiano, B. et al. Modelling lagrangian velocity and acceleration in turbulent flows as infinitely differentiable stochastic processes. *J. Fluid Mech.* **900**, A27 (2020).

[21] Lamorgese, A., Pope, S. B., Yeung, P. & Sawford, B. L. A conditionally cubic-gaussian stochastic lagrangian model for acceleration in isotropic turbulence. *J. Fluid Mech.* **582**, 423–448 (2007).

[22] Minier, J.-P., Chibbaro, S. & Pope, S. B. Guidelines for the formulation of lagrangian stochastic models for particle simulations of single-phase and dispersed two-phase turbulent flows. *Phys. Fluids* **26**, 113303 (2014).

[23] Wilson, J. D. & Sawford, B. L. Review of lagrangian stochastic models for trajectories in the turbulent atmosphere. *Bound.-Layer. Meteorol.* **78**, 191–210 (1996).

[24] Bourlioux, A., Majda, A. & Volkov, O. Conditional statistics for a passive scalar with a mean gradient and intermittency. *Phys. Fluids*10.1063/1.2353880 (2006).

[25] Majda, A. J. & Gershgorin, B. Elementary models for turbulent diffusion with complex physical features: eddy diffusivity, spectrum and intermittency. *Philos. Trans. R. Soc. A* **371**, 20120184 (2013). 10.1098/rsta.2012.0184 23185058

[26] Biferale, L., Boffetta, G., Celani, A., Crisanti, A. & Vulpiani, A. Mimicking a turbulent signal: sequential multiaffine processes. *Phys. Rev. E* **57**, R6261 (1998).

[27] Arneodo, A., Bacry, E. & Muzy, J.-F. Random cascades on wavelet dyadic trees. *J. Math. Phys.* **39**, 4142–4164 (1998).

[28] Bacry, E. & Muzy, J. F. Log-infinitely divisible multifractal processes. *Commun. Math. Phys.* **236**, 449–475 (2003).

[29] Chevillard, L. et al. On a skewed and multifractal unidimensional random field, as a probabilistic representation of Kolmogorov’s views on turbulence. *Ann. Henri Poincaré* **20**, 3693–3741 (2019).

[30] Sinhuber, M., Friedrich, J., Grauer, R. & Wilczek, M. Multi-level stochastic refinement for complex time series and fields: a data-driven approach. *N. J. Phys.* **23**, 063063 (2021).

[31] Lübke, J., Friedrich, J. & Grauer, R. Stochastic interpolation of sparsely sampled time series by a superstatistical random process and its synthesis in fourier and wavelet space. *J. Phys.: Complex.* **4**, 015005 (2022).

[32] Zamansky, R. Acceleration scaling and stochastic dynamics of a fluid particle in turbulence. *Phys. Rev. Fluids* **7**, 084608 (2022).

[33] Arnéodo, A. et al. Universal intermittent properties of particle trajectories in highly turbulent flows. *Phys. Rev. Lett.* **100**, 254504 (2008). 18643666 10.1103/PhysRevLett.100.254504

[34] Kingma, D. P. & Welling, M. Auto-Encoding Variational Bayes. In *2nd International Conference on Learning Representations: Conference Track Proceedings* (ICLR, 2014); 10.48550/arXiv.1312.6114

[35] Goodfellow, I. et al. Generative adversarial nets. *Adv. Neural Infor. Process. Syst.* **27**, 2672–2680 (2014).

[36] Ho, J., Jain, A. & Abbeel, P. Denoising diffusion probabilistic models. *Adv. Neural Inf. Process. Syst.* **33**, 6840–6851 (2020).

[37] Dhariwal, P. & Nichol, A. Diffusion models beat gans on image synthesis. *Adv. Neural Inf. Process. Syst.* **34**, 8780–8794 (2021).

[38] van den Oord, A. et al. WaveNet: a generative model for raw audio. Preprint at 10.48550/arXiv.1609.03499 (2016).

[39] Brown, T. et al. Language models are few-shot learners. *Adv. Neural Inf. Process. Syst.* **33**, 1877–1901 (2020).

[40] Chen, R. J., Lu, M. Y., Chen, T. Y., Williamson, D. F. & Mahmood, F. Synthetic data in machine learning for medicine and healthcare. *Nat. Biomed. Eng.* **5**, 493–497 (2021). 34131324 10.1038/s41551-021-00751-8 PMC9353344

[41] Duraisamy, K., Iaccarino, G. & Xiao, H. Turbulence modeling in the age of data. *Annu. Rev. Fluid Mech.* **51**, 357–377 (2019).

[42] Brunton, S. L., Noack, B. R. & Koumoutsakos, P. Machine learning for fluid mechanics. *Annu. Rev. Fluid Mech.* **52**, 477–508 (2020).

[43] Vlachas, P. R., Byeon, W., Wan, Z. Y., Sapsis, T. P. & Koumoutsakos, P. Data-driven forecasting of high-dimensional chaotic systems with long short-term memory networks. *Proc. R. Soc. A* **474**, 20170844 (2018). 29887750 10.1098/rspa.2017.0844 PMC5990702

[44] Pathak, J., Hunt, B., Girvan, M., Lu, Z. & Ott, E. Model-free prediction of large spatiotemporally chaotic systems from data: A reservoir computing approach. *Phys. Rev. Lett.* **120**, 024102 (2018). 29376715 10.1103/PhysRevLett.120.024102

[45] Mohan, A. T., Tretiak, D., Chertkov, M. & Livescu, D. Spatio-temporal deep learning models of 3d turbulence with physics informed diagnostics. *J. Turbul.* **21**, 484–524 (2020).

[46] Kim, J. & Lee, C. Deep unsupervised learning of turbulence for inflow generation at various reynolds numbers. *J. Comput. Phys.* **406**, 109216 (2020).

[47] Guastoni, L. et al. Convolutional-network models to predict wall-bounded turbulence from wall quantities. *J. Fluid Mech.* **928**, A27 (2021).

[48] Buzzicotti, M., Bonaccorso, F., Di Leoni, P. C. & Biferale, L. Reconstruction of turbulent data with deep generative models for semantic inpainting from turb-rot database. *Phys. Rev. Fluids* **6**, 050503 (2021).

[49] Yousif, M. Z., Yu, L., Hoyas, S., Vinuesa, R. & Lim, H. A deep-learning approach for reconstructing 3d turbulent flows from 2d observation data. *Sci. Rep.* **13**, 2529 (2023). 36781944 10.1038/s41598-023-29525-9 PMC9925827

[50] Shu, D., Li, Z. & Farimani, A. B. A physics-informed diffusion model for high-fidelity flow field reconstruction. *J. Comput. Phys.* **478**, 111972 (2023).

[51] Buzzicotti, M. Data reconstruction for complex flows using AI: recent progress, obstacles, and perspectives. *Europhys. Lett.* **142**, 23001 (2023).

[52] Granero-Belinchon, C. Neural network based generation of a 1-dimensional stochastic field with turbulent velocity statistics. *Phys. D* **458**, 133997 (2024).

[53] Nichol, A. Q. & Dhariwal, P. Improved denoising diffusion probabilistic models. In *International Conference on Machine Learning* (eds. Meila, M. et al.) 8162–8171 (PMLR, 2021).

[54] Chevillard, L. et al. Lagrangian velocity statistics in turbulent flows: effects of dissipation. *Phys. Rev. Lett.* **91**, 214502 (2003). 14683309 10.1103/PhysRevLett.91.214502

[55] Biferale, L. et al. Multifractal statistics of lagrangian velocity and acceleration in turbulence. *Phys. Rev. Lett.* **93**, 064502 (2004). 15323634 10.1103/PhysRevLett.93.064502

[56] Sohl-Dickstein, J., Weiss, E., Maheswaranathan, N. & Ganguli, S. Deep unsupervised learning using nonequilibrium thermodynamics. In *International Conference on Machine Learning*, 2256–2265 (PMLR, 2015).

[57] Burda, Y., Grosse, R. & Salakhutdinov, R. Accurate and conservative estimates of mrf log-likelihood using reverse annealing. In *Artificial Intelligence and Statistics*, 102–110 (PMLR, 2015).

[58] Mordant, N., Delour, J., Léveque, E., Arnéodo, A. & Pinton, J.-F. Long time correlations in lagrangian dynamics: a key to intermittency in turbulence. *Phys. Rev. Lett.* **89**, 254502 (2002). 12484891 10.1103/PhysRevLett.89.254502

[59] Angriman, S., Mininni, P. D. & Cobelli, P. J. Multitime structure functions and the lagrangian scaling of turbulence. *Phys. Rev. Fluids* **7**, 064603 (2022).

[60] Mitra, D. & Pandit, R. Varieties of dynamic multiscaling in fluid turbulence. *Phys. Rev. Lett.* **93**, 024501 (2004). 15323922 10.1103/PhysRevLett.93.024501

[61] L’vov, V. S., Podivilov, E. & Procaccia, I. Temporal multiscaling in hydrodynamic turbulence. *Phys. Rev. E* **55**, 7030 (1997).

[62] Borgas, M. The multifractal lagrangian nature of turbulence. *Philos. Trans. R. Soc. Lond. Ser. A* **342**, 379–411 (1993).

[63] Nelkin, M. Multifractal scaling of velocity derivatives in turbulence. *Phys. Rev. A* **42**, 7226 (1990). 9904037 10.1103/physreva.42.7226

[64] Paladin, G. & Vulpiani, A. Degrees of freedom of turbulence. *Phys. Rev. A* **35**, 1971 (1987). 10.1103/physreva.35.1971 9898371

[65] Meneveau, C. Transition between viscous and inertial-range scaling of turbulence structure functions. *Phys. Rev. E* **54**, 3657 (1996). 10.1103/physreve.54.3657 9965514

[66] Benzi, R. et al. A random process for the construction of multiaffine fields. *Phys. D* **65**, 352–358 (1993).

[67] Guth, F., Coste, S., De Bortoli, V. & Mallat, S. Wavelet score-based generative modeling. *Adv. Neural Inf. Process. Syst.* **35**, 478–491 (2022).

[68] Salazar, J. P. & Collins, L. R. Two-particle dispersion in isotropic turbulent flows. *Annu. Rev. Fluid Mech.* **41**, 405–432 (2009).

[69] Scatamacchia, R., Biferale, L. & Toschi, F. Extreme events in the dispersions of two neighboring particles under the influence of fluid turbulence. *Phys. Rev. Lett.* **109**, 144501 (2012). 23083247 10.1103/PhysRevLett.109.144501

[70] Biferale, L. et al. Multiparticle dispersion in fully developed turbulence. *Phys. Fluids* **17**, 111701 (2005).

[71] Xu, H., Pumir, A. & Bodenschatz, E. The pirouette effect in turbulent flows. *Nat. Phys.* **7**, 709–712 (2011).

[72] Roemmich, D. et al. On the future of argo: a global, full-depth, multi-disciplinary array. *Front. Mar. Sci.* **6**, 439 (2019).

[73] Essink, S., Hormann, V., Centurioni, L. R. & Mahadevan, A. On characterizing ocean kinematics from surface drifters. *J. Atmos. Ocean. Technol.* **39**, 1183–1198 (2022).

[74] Biferale, L., Buzzicotti, M., Bonaccorso, F. & Calascibetta, C. Turb-lagr. a database of 3d lagrangian trajectories in homogeneous and isotropic turbulence. Preprint at 10.48550/arXiv.2303.08662 (2023).

[75] Calascibetta, C., Biferale, L. & Borra, F. et al. Optimal tracking strategies in a turbulent flow. *Commun. Phys.* **6**, 256 (2023).

[76] Van Hinsberg, M., Thije Boonkkamp, J., Toschi, F. & Clercx, H. On the efficiency and accuracy of interpolation methods for spectral codes. *SIAM J. Sci. Comput.* **34**, B479–B498 (2012).

[77] Ronneberger, O., Fischer, P. & Brox, T. U-net: convolutional networks for biomedical image segmentation. In *Medical Image Computing and Computer-Assisted Intervention* (eds. Navab, N., et al.) 234–241 (Springer, 2015).

[78] Vaswani, A. et al. Attention is all you need. *Adv. Neural Inf. Process. Syst.* **30**, 5998–6008 (2017).

[79] Song, J., Meng, C. & Ermon, S. Denoising diffusion implicit models. In *International Conference on Learning Representations* (2021); https://openreview.net/forum?id=St1giarCHLP

[80] Lu, C. et al. Dpm-solver: a fast ode solver for diffusion probabilistic model sampling in around 10 steps. *Adv. Neural Inf. Process. Syst.* **35**, 5775–5787 (2022).

[81] Li, T., Biferale, L., Bonaccorso, F., Scarpolini, M. A. & Buzzicotti, M. Dataset for: Synthetic lagrangian turbulence by generative diffusion models. *INFN*10.15161/oar.it/143615 (2024). 10.1038/s42256-024-00810-0 PMC12151855 40510238

[82] Smartturb/diffusion-lagr: stable. *Zenodo*10.5281/zenodo.10563386 (2024).

[83] Li, T., Biferale, L., Bonaccorso, F., Scarpolini, M. A. & Buzzicotti, M. Supplementary code for: Synthetic lagrangian turbulence by generative diffusion models. *CodeOcean* https://codeocean.com/capsule/0870187/tree/v1 (2024). 10.1038/s42256-024-00810-0 PMC12151855 40510238

[84] Mordant, N., Lévêque, E. & Pinton, J.-F. Experimental and numerical study of the lagrangian dynamics of high reynolds turbulence. *N. J. Phys.* **6**, 116 (2004).

[85] Homann, H., Grauer, R., Busse, A. & Müller, W.-C. Lagrangian statistics of navier–stokes and mhd turbulence. *J. Plasma Phys.* **73**, 821–830 (2007).

[86] Biferale, L., Boffetta, G., Celani, A., Lanotte, A. & Toschi, F. Particle trapping in three-dimensional fully developed turbulence. *Phys. Fluids* **17**, 021701 (2005).

[87] Fisher, R. T. et al. Terascale turbulence computation using the flash3 application framework on the ibm blue gene/l system. *IBM J. Res. Dev.* **52**, 127–136 (2008).

[88] Yeung, P., Pope, S. B. & Sawford, B. L. Reynolds number dependence of lagrangian statistics in large numerical simulations of isotropic turbulence. *J. Turbul.* **7**, N58 (2006).

[89] Xu, H., Bourgoin, M., Ouellette, N. T. & Bodenschatz, E. et al. High order lagrangian velocity statistics in turbulence. *Phys. Rev. Lett.* **96**, 024503 (2006). 16486587 10.1103/PhysRevLett.96.024503

[90] Berg, J., Lüthi, B., Mann, J. & Ott, S. Backwards and forwards relative dispersion in turbulent flow: an experimental investigation. *Phys. Rev. E* **74**, 016304 (2006). 10.1103/PhysRevE.74.016304 16907188

