# Supplementary material for: Synthetic Lagrangian turbulence by generative diffusion models
Source: Nat Mach Intell. 2024 Apr 17;6(4):393–403. doi: 10.1038/s42256-024-00810-0 (PMC12151855; doi:10.1038/s42256-024-00810-0)
Supplement: Supplementary file 1 — Supplementary Figs. 1 and 2 and Discussion. [file 42256_2024_810_MOESM1_ESM.pdf]

nature machine intelligence

Article

https://doi.org/10.1038/s42256-024-00810-0

Synthetic Lagrangian turbulence by generative diffusion models

In the format provided by the authors and unedited

Contents

Effects of Training Data Amount

Wasserstein Generative Adversarial Networks

Effects of Training Data Amount. In Fig. 1 we present statistical measurements derived from two DM-1c models trained under different conditions: DM-1c-10% and DM-1c-1%, each using only 10% and 1% of the DNS data, respectively. The results show that the DM-1c-10% model closely matches the full DM-1c, showing a consistent agreement with the DNS data. However, when we push the boundary further by drastically reducing the training data, the DM-1c-1% model shows significant deviation from the DNS benchmarks. This stark contrast reveals the lower limit of training data required for Diffusion Models (DMs) to accurately extrapolate extreme events.

Wasserstein Generative Adversarial Networks. To compare the performance of DM against a different generative model, we have implemented and tested a Wasserstein Generative Adversarial Network (WGAN) [1], which is an improved variant of the standard generative adversarial network (GAN), generally leading to more stable training [2]. The main advantage of GANs over DMs is that they map the input noise into the generated data in a single prediction step, resulting in a much faster generation. The generator, G, in the WGAN aims to learn how to generate samples that satisfy the distribution of the ground truth trajectories, $q(\mathcal{V}(t))$, by minimizing the loss provided by the discriminator, D, defined as:

$$L_G = \mathbb{E}_q D(\mathcal{V}) - \mathbb{E}_{p_\theta} D(\mathcal{V}), \tag{1}$$

where the first term represents the average over the ground truth data, while the second mean is obtained by sampling from the generated trajectories. As defined, L_G corresponds to the Wasserstein distance between the true and generated probability distributions, measured by D, which is simultaneously trained to ensure the correct evaluation of this distance by minimizing the following loss,

$$L_D = \underbrace{-\left(\mathbb{E}_q D(\mathcal{V}) - \mathbb{E}_{p_\theta} D(\mathcal{V})\right)}_{\text{-}L_G} + \underbrace{\lambda\left(\mathbb{E}_{p_\theta}[(||\nabla_{\mathcal{V}} D(\mathcal{V})||_2 - 1)^2]\right)}_{\text{gradient penalty}}. \tag{2}$$

The first term in the discriminator's loss, optimizes the estimation of the Wasserstein distance between q and p_θ, while the second term acts as a gradient penalty to enforce the 1-Lipschitz constraint, as required by the definition of the Wasserstein distance [3], λ is a hyperparameter set to 10 as in [2]. To ensure the accuracy of the discriminator, which is fundamental in this optimization process, we performed many backpropagation steps (30 steps) on the discriminator weights before each update of the generator network. Both the discriminator and the generator networks consist of a series of 1D convolutional layers, each with batch normalization and ReLU activation function. In particular, the generator starts with a fully connected layer that connects the random noise in the input to the sequence of 1D convolutional layers configured to progressively increase the length of the time signal. The discriminator is specular and ends with a fully connected layer that provides a scalar value at the output, i.e. the Wasserstein distance. In Fig. 2, we have compared the results of DNS with those of WGAN-1c, which refers to the WGAN model specifically trained on a single component of the Lagrangian velocity. Despite the improved convergence properties of WGAN with respect to the standard GAN, it does not perform as well as the DM model, especially concerning the transition between the inertial-range behavior and that at dissipative scales around τ_η where we observe the larger deviations from the scaling of the original data. It is worth noting that unless one needs to generate very accurate samples, WGAN is much faster in sampling time and could be the tool of choice in many applications.

[1] Arjovsky, M., Chintala, S. & Bottou, L. Wasserstein generative adversarial networks. In *International conference on machine learning*, 214–223 (PMLR, 2017).

[2] Gulrajani, I., Ahmed, F., Arjovsky, M., Dumoulin, V. & Courville, A. C. Improved training of wasserstein gans. *Advances in neural information processing systems* **30** (2017).

[3] Villani, C. The wasserstein distances. *Optimal Transport: Old and New* 93–111 (2009).

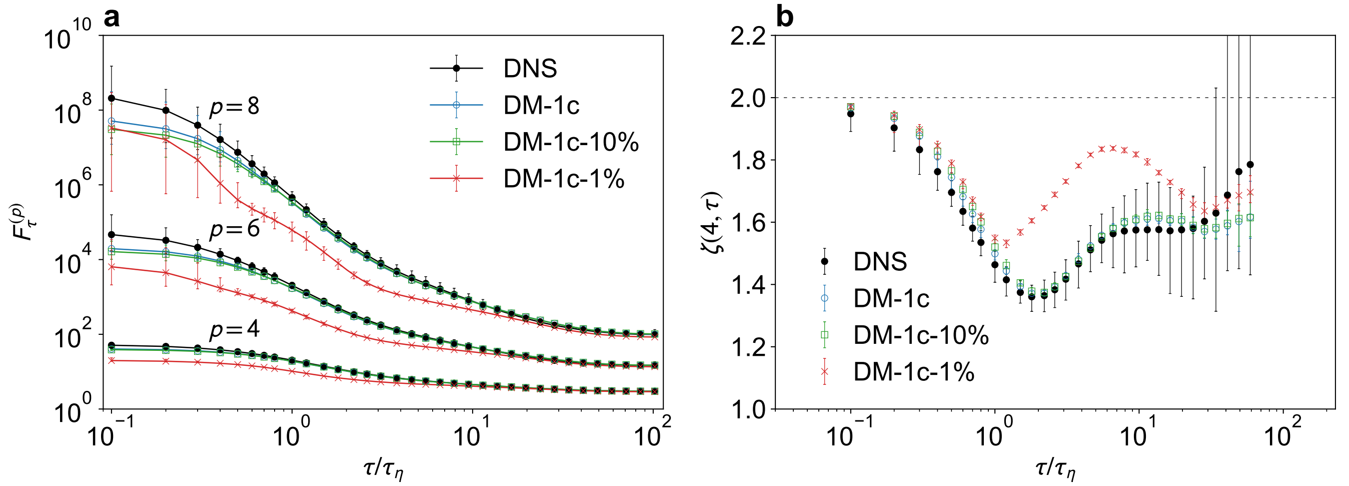

FIG. 1. **Scale-by-scale statistical properties.** **a**, Log-log plot of the generalized flatness, $F_\tau^{(p)}$, for $p = 4, 6$ and 8, compared across DNS, DM-1c, DM-1c-10%, and DM-1c-1%. **b**, Comparison between the ground-truth DNS and the three DM-1c models, on the lin-log scale, for the 4th-order logarithmic local slope $\zeta(4,\tau)$. The error bars represent the minimum and maximum values obtained for each measure by dividing the entire data set used to compute the statistics into 10 different independent batches of smaller size. Error bars may appear smaller than the data points.

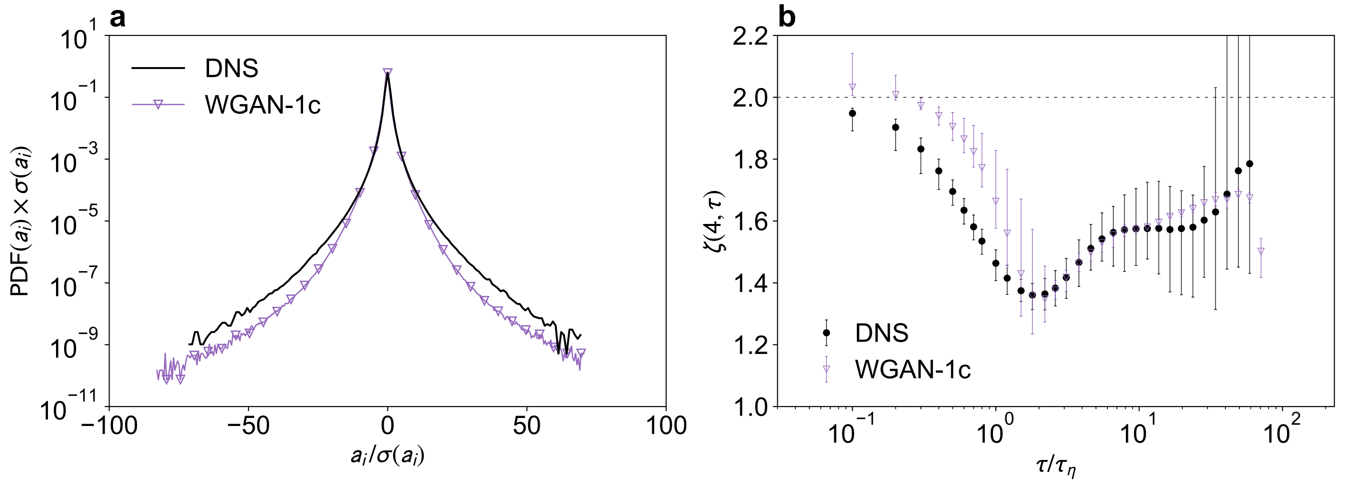

FIG. 2. **WGAN and DNS statistical comparison.** **a**, Standardized PDFs of one generic component of the acceleration, a_i, for both ground-truth DNS data (black line) and synthetically generated data from WGAN-1c (purple line with triangles). **b**, Scale-by-scale statistical comparison between the ground-truth DNS and the WGAN-1c results, on the lin-log scale, for the 4th-order logarithmic local slope $\zeta(4,\tau)$. Statistics and error bars are derived as in Fig. 1.
